# Supplementary material for: Fauna associated with shallow-water methane seeps in the Laptev Sea
Source: PeerJ. 2020 May 4;8:e9018. doi: 10.7717/peerj.9018 (PMC7204824; doi:10.7717/peerj.9018)
Supplement: Supplemental Information 2 [file peerj-08-9018-s002.docx]

UNCTREE

Unconstrained divisive cluster

*Resemblance worksheet*

Name: Resem3

Data type: Similarity

Selection: All

*Parameters*

Minimum group size: 1

Minimum split size: 2

Minimum split R: 0

Simprof test

*Data worksheet*

Name: Data6

Data type: Biomass

Sample selection: All

Variable selection: All

*Simprof Parameters*

Type 1 (Analyse: Samples - Permute within: Variables)

Number of permutations: 999

Significance level: 5%

Resemblance:

Resemblance measure: S17 Bray-Curtis similarity

A->B,D

Pi: 3.12 Sig(%): 0.1

R: 0.68 B%: 86.5

B->(12),C

Pi: 1.97 Sig(%): 58.2

R: 1 B%: 10.1

C->(11),(10)

R: 0 B%: 0

D->E,I

Pi: 2.79 Sig(%): 0.1

R: 0.58 B%: 63.9

E->F,G

Pi: 2.45 Sig(%): 12.2

R: 0.92 B%: 37.4

F->(18),(16)

R: 0 B%: 0

G->(6),H

R: 1 B%: 6.8

H->(5),(4)

R: 0 B%: 0

I->J,K

Pi: 3.3 Sig(%): 0.1

R: 0.67 B%: 64.7

J->(1),(2)

Pi: 0 Sig(%): 100

R: 0 B%: 0

K->L,Q

Pi: 2.66 Sig(%): 0.1

R: 0.67 B%: 37.8

L->(9),M

Pi: 2.19 Sig(%): 10.1

R: 0.96 B%: 29

M->N,O

R: 1 B%: 6.9

N->(19),(20)

R: 0 B%: 0

O->P,(21)

R: 1 B%: 2.2

P->(8),(7)

R: 0 B%: 0

Q->R,T

Pi: 2.32 Sig(%): 16.9

R: 0.75 B%: 26

R->S,(13)

R: 1 B%: 13.6

S->(17),(14)

R: 0 B%: 0

T->(3),(15)

R: 0 B%: 0

*Outputs*

Plot: Graph11
