# Supplementary material for: Fauna associated with shallow-water methane seeps in the Laptev Sea
Source: PeerJ. 2020 May 4;8:e9018. doi: 10.7717/peerj.9018 (PMC7204824; doi:10.7717/peerj.9018)
Supplement: Supplemental Information 3 — Mean values of abundance, biomass, species number per grab, Pielou evenness, Hurlbert rarefaction index for 100 individuals and Shannon-Wiener index (H’ ln) with standard deviation are shown. [file peerj-08-9018-s003.docx]

Diversity characteristics in station groups. Mean values of abundance, biomass, species number per grab, Pielou evenness, Hurlbert rarefaction index for 100 individuals and Shannon-Wiener index (H’ ln) with standard deviation are shown.

| **Station group** | **Density (ind. m^-2^)** | **Biomass (g ww m^-2^)** | **Species number** | **Pielou evenness** | **ES(100)** | **Shannon-Wiener index** |
| --- | --- | --- | --- | --- | --- | --- |
| *Control* | 680 ±92 | 87.35 ±50.00 | 24 ±8 | 0.85 ±0.03 | 23.67 ±2.31 | 2.69 ±0.05 |
| *C15 background* | 1996 ±202 | 26.58 ±7.78 | 40 ±6 | 0.81 ±0.03 | 29.49 ±3.33 | 2.97 ±0.12 |
| *C15-seep a* | 1996 ±851 | 37.53 ±9.33 | 34 ±9 | 0.71 ±0.24 | 25.36 ±7.30 | 2.52 ±0.9 |
| *С15-seep b* | 7625 ±3189 | 39.36 ±32.64 | 40 ±14 | 0.55 ±0.13 | 18.63 ±4.92 | 2.04 ±0.68 |
| *Oden* | 3112 ±679 | 155.16 ±126.42 | 52 ±4 | 0.75 ±0.04 | 30.09 ±1.06 | 2.95 ±0.16 |
